# Supplementary material for: Microcomb-based integrated photonic processing unit
Source: Nat Commun. 2023 Jan 5;14:66. doi: 10.1038/s41467-022-35506-9 (PMC9814295; doi:10.1038/s41467-022-35506-9)
Supplement: Supplementary file 1 — Supplementary Information [file 41467_2022_35506_MOESM1_ESM.pdf]

Supplementary Information for

Microcomb-based integrated photonic processing unit

Bowen Bai[1,*], Qipeng Yang[1,*], Haowen Shu[1,*], Lin Chang[2,1,3,*,†], Fenghe Yang[4], Bitao Shen[1], Zihan Tao[1], Jing Wang[5], Shaofu Xu[5], Weiqiang Xie[2], Weiwen Zou[5], Weiwei Hu[1], John E. Bowers[2,†] and Xingjun Wang[1,3,6,†]

[1]State Key Laboratory of Advanced Optical Communications System and Networks, School of Electronics, Peking University, Beijing, 100871, China.

[2]Department of Electrical and Computer Engineering, University of California, Santa Barbara, CA 93106, USA.

[3]Frontiers Science Center for Nano-optoelectronics, Peking University, Beijing 100871, China.

[4]Zhangjiang Laboratory, Shanghai, 201210, China.

[5]State Key Laboratory of Advanced Optical Communications System and Networks, Department of Electronic Engineering, Shanghai Jiao Tong University, Shanghai, 200240, China.

[6]Peking University Yangtze Delta Institute of Optoelectronics, Nantong 226010, China.

[*]These authors contributed equally to this work.

Corresponding authors: [†]linchang@pku.edu.cn, [†]bowers@ece.ucsb.edu, [†]xjwang@pku.edu.cn.

Supplementary note 1: Characterization of the silicon photonic chip

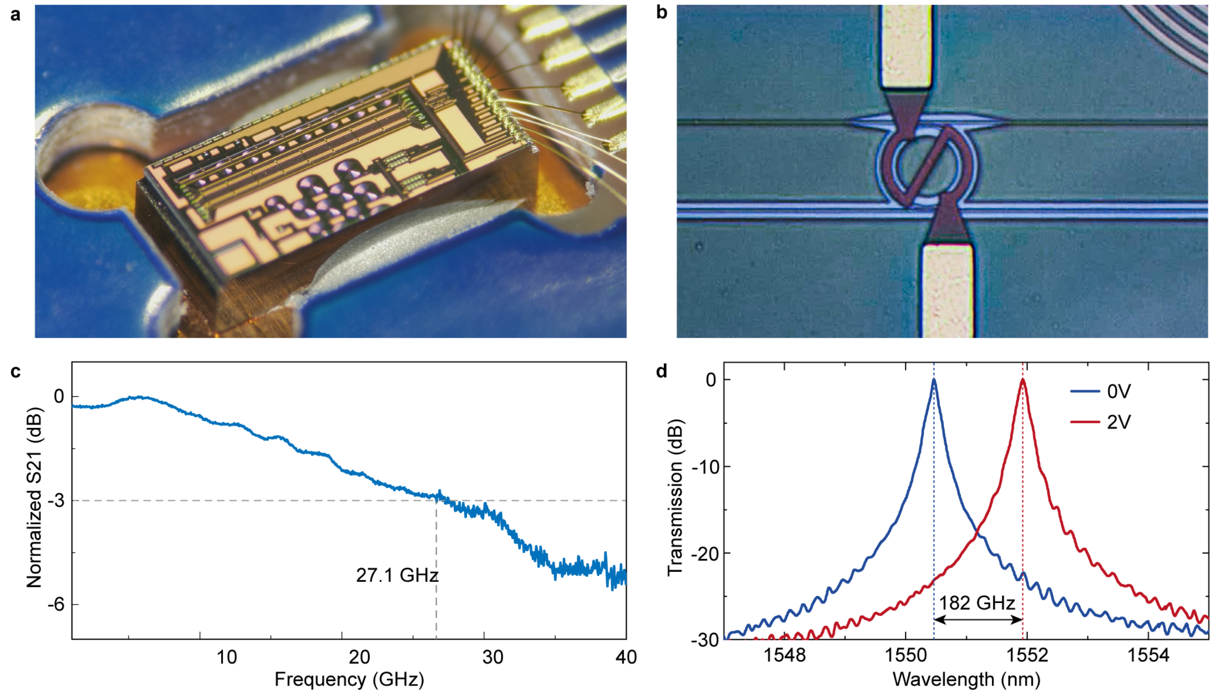

Fig. 1: characteristics of the silicon EOM and one MRR with on-ring heate. a, The photograph of packaged chip. **b**, Optical image of one MRR. **c**, Measured bandwidth of the silicon EO modulator. **d**, The transmission spectrum of one MRR.

The packaged silicon photonic chip is illustrated in Fig. S1a. The 12 pads with space of 100 μm for thermal tuning is bonded to the PCB using golden wires. The 5 signal pads (GSGSG) with space of 100 μm is connected to the AWG via high frequency probe when conducting the experiment. The silicon EOM works under the carrier-depletion mode and the measured 3 dB EO bandwidth is above 27 GHz, as indicated in Fig. S1c. Therefore, the modulation rate of our PPU can be further improved by reducing the time delay step. The insertion loss of the silicon EOM is about 5 dB.

Kernel weight is tuned using thermo-optic effect. The MRR with on-ring TiN heater is shown in Fig. S1b. The quality factor (Q) of the MRR is about 22000 and the width of the TiN heater is 2 μm. The voltage required to drive the heater for a 1.47 nm (182 GHz) wavelength shift is less than 2 V, as Fig. S1d shows. Although the maximum tuning frequency is only 10 kHz, once the kernel weight is loaded onto the MRR, the heater do not require tuning anymore in a short time.

Supplementary note 2: Inner cross-talk in the MRR weight bank

Three kinds of inner cross-talk exist in the MRR weight bank: 1) transmission spectrum overlap; 2) thermal leakage; 3) parasitic resistance. In our design, the channel spacing is 182 GHz, which is wide enough to effectively inhibit the MRR spectrum overlap. Thermal crosstalk happens when the local heat leaks to the surroundings during operation, leading to resonance shifts in the nearby MRRs. Although thermo-electric cooler (TEC) are valid approaches to reduce the thermal crosstalk, the thermal crosstalk still exist when tuning other channels.

Parasitic resistance is a sort of electrical crosstalk which usually occurs as the on-ring heaters share a common ground. In multichannel cases, the parasitic resistance should be taken into consideration. Here, we abstracts the MRR weight bank into a simple electrical model: n TiN on-ring heaters with resistance of R_{ring} are connected to the common ground. The effective resistance of the ground trace is R_g. The applied voltage on the m-th channel can be expressed as

$$V_m = R_{ring} i_m + R_g \sum_{m=0}^{n-1} i_m = \hat{V}_m + \Delta V_m, \tag{1}$$

where i_m is the current pass through the R_{ring}, $\hat{V}_m$ is the actual voltage across the m-th on-ring heater. ΔV_m is the electrical crosstalk from all channels due to the non-zero common ground resistance. Since the resonance state of the MRR, or rather, kernel weight depend on the $\hat{V}_m$, the electrical crosstalk from parasitic resistance needs to be considered and eliminated.

Supplementary note 3: Calibration of the MRR weight bank

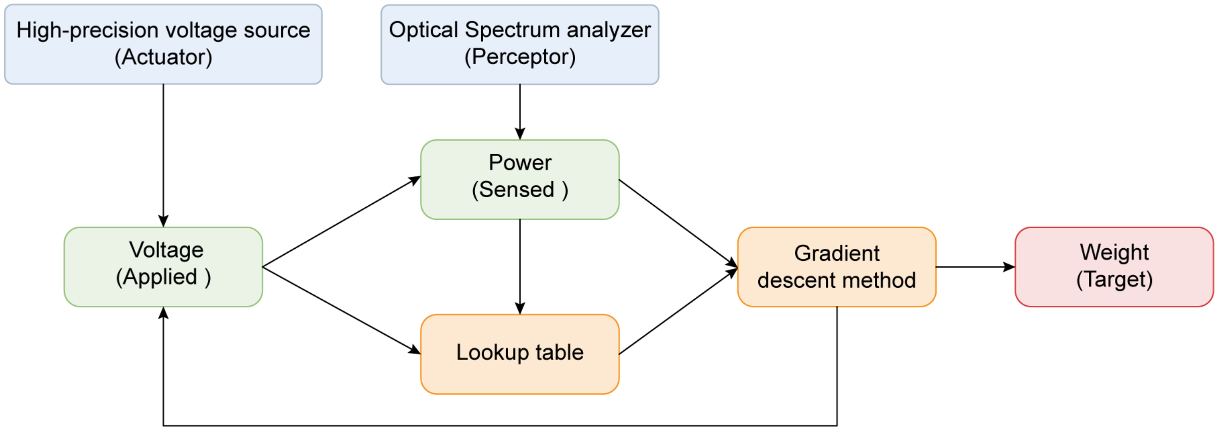

Fig. 2: Calibration flow chart using *in-situ* GDC method. Blue: physical interface. Green: physical parameters can be controlled or sensed. Orange: submethods used for *in-situ* GDC. Red: parameters needed to be calibrated.

In order to obtain accurate convolution results, the accuracy of the weights loading using MRRs is essential. In this work, we face three factors that affect the MRR resonance state: (1) transmission spectrum overlap (2) thermal leakage (3) parasitic resistance. In order to solve the adverse effects of these three factors, we combine the lookup table with *in-situ* GDC to correct the weights loading.

The calibration procedure for MRR weight bank using *in-situ* GDC method is shown Fig. S2. During the calibration, we use high-precision voltage sources to control the resonance states of the MRRs and an optical spectrum analyzer to read the power of each channel. Appropriate voltage range is chosen for each channel to ensure that the lookup table contains normalized weights from 0-1. Then, we increase the voltage with one small step (0.01V), which makes the spectrum start to shift. The optical power at the corresponding channel is read through optical spectrum analyzer. After voltage sweeping, we normalize the recorded optical power to link the applied voltage with normalized power (weight).

However, the inner cross-talk will degenerate the accuracy of the static lookup table during the weight mapping. When other channels are set with a weight list of all combination of (0,0.33,0.66,1), the weights of the given will deviate from the target ones. Fortunately, these errors can be reduced by *in-situ* GDC. In the calibration, the lookup table of the given channel provides the initial voltage of the target weight, which dramatically shorten the

time for *in-situ* GDC. The calibration starts from the initial voltage and sequentially uses gradient descent method to update the voltage values. Here, we use finite difference method to calculate the gradient. The formula is shown in the main text equation (5). In order to ensure the loss function (defined in the main text equation (4)) quickly converges, we need to select appropriate learning rate according to the target weight. When the learning rate and gradient are determined, the voltage is updated according to the equation (6) in the main text until the difference between the actual weight and the target weight value is less than 0.001. It should be noted that the above procedure requires multiple training epochs, because the calibration for a given channel affects the other calibrated channels. As the number of training epochs increases, the effects of inner cross-talk gradually decreases. The *in-situ* GDC can only be terminated when the difference between the actual and the target weight of all channels is less than 0.001.

To evaluate our calibration procedure quantificationally, the concept of weight control precision is introduced. The weight control precision generally denotes the number of discrete levels that can be reached. For example, 8-bit precision indicates 2^8=256 discrete levels that can be achieved. In our photonic MRR weight bank, assume the measured weight is w and the target weight is $\hat{w}$, then the number of discrete levels or the dynamic range can be expressed as

$$N = \frac{w_{\max} - w_{\min}}{2|w - \hat{w}|_{\max}}, \tag{2}$$

where $|w - \hat{w}|_{\max}$ refers to the maximum value of the deviation from the command weights. Given that the deviation can be positive or negative, the minimum weight interval that can be distinguished is $2|w - \hat{w}|_{\max}$. In our case, $w_{\max}$=1, $w_{\min}$=0, $|w - \hat{w}|_{\max} \approx 0.001$, then the effective bit resolution can be written as

$$\log_2(N) = \log_2\left(\frac{1-0}{2 \times 1 \times 10^{-3}}\right) \approx 9\text{bits} \tag{3}$$

It should be noted that if using the same calculation method in Ref.[1], our weight control accuracy can be as high as 9.97 bits.

Supplementary note 4: Time delay measurement

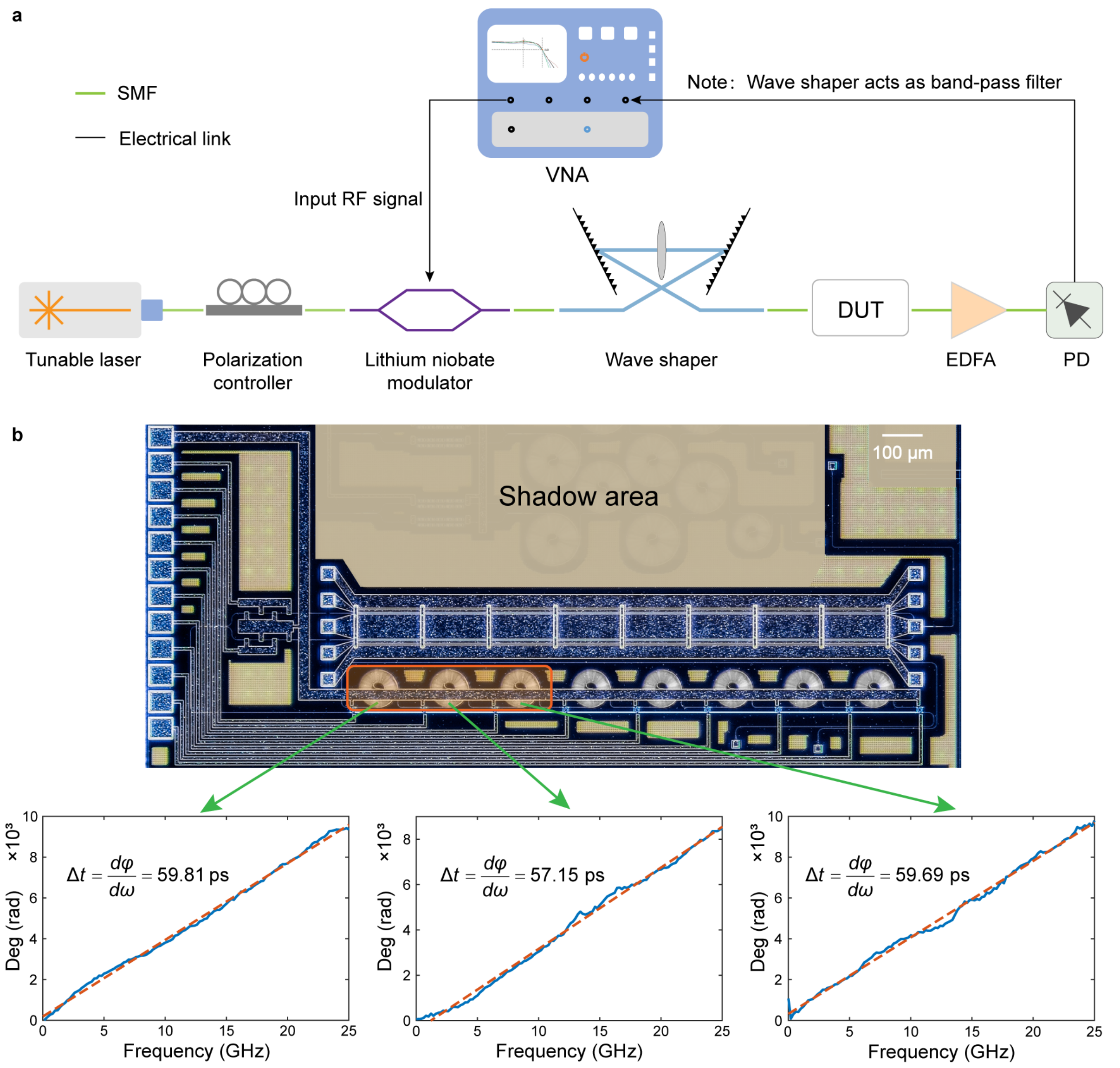

Fig. 3: Time delay measurement. a, Experiment setup. **b**, Measured results for each on-chip delay line.

Since the time delay step directly determines the modulation rate, the measurement of the delay time introduced by the on-chip delay lines is necessary. Here, we conduct a experiment to measure the group delay of the optical path for each channel.

Fig. S3a shows the experiment setup for time delay measurement. For the i-th channel, the resonant frequency of the MRR is f_i and the frequency of the optical carrier from a tunable laser (EXFO T100S-HP) is f_{ci} (slightly larger than f_i). The optical carrier is

modulated by a lithium niobate intensity modulator (EOspace, 35 GHz bandwidth) to yield an optical double-sideband (ODSB) signal. The input RF signal with a frequency of f_m is from a vector network analyzer (VNA, Keysight N5247A). The ODSB optical signal propagates to a wave shaper (Finisar Waveshaper 4000s), which acts as a tunable band-pass filter. The left sidband of the ODSB signal is wipe off while the optical carrier and right sideband are retained, and therefore, the ODSB signal turns into optical single sideband (OSSB) signal. The OSSB signal goes to the device-under-test (DUT) and experiences phase changes due to the frequency response of the DUT at $f_{ci}+f_m$. Finally, the optical signal with phase variation is amplified by a low-noise erbium-doped fiber amplifier (Amonics AEDFA-PA-35-B-FA) and converted to electrical signal by a fast photodetector (PD, FINISAR HPDV2120R).Since the electrical response at f_m represents the optical response of the DUT at $f_{ci}+f_m$, by sweeping the frequency of the RF signal from VNA, the optical phase response of the DUT can be obtained[2].

In our measurement, the frequency f_m varied from 0 to 25 GHz and the wavelength of the optical carrier λ_{ci} is set to slightly less than the resonant wavelength of the i-th MRR λ_i. Thus, the right sideband of the OSSB signal could go through the passband of the MRR. Assume the group delay of i-th channel is tg_i, the delay time between $i+1$-th and i-th channel is written as

$$\Delta t_i = tg_{i+1} - tg_i = \frac{d\varphi_i}{d\omega}, i = 0, 1, 2, \cdots, \mathrm{n}-1 \tag{4}$$

where the φ_i is the accumulation of the phase variation for i-th channel, ω is the angular frequency and n is the number of the channels. Fig. S3b gives the measured delay time between adjacent channels. Then, fhe delay time step Δt is defined as

$$\Delta t = \frac{1}{n}\sum_{i=0}^{n-1} \Delta t_i = \frac{1}{3}\left(\Delta t_0 + \Delta t_1 + \Delta t_2\right) = 58.88\mathrm{ps} \tag{5}$$

Supplementary note 5: Roberts operator for edge detection

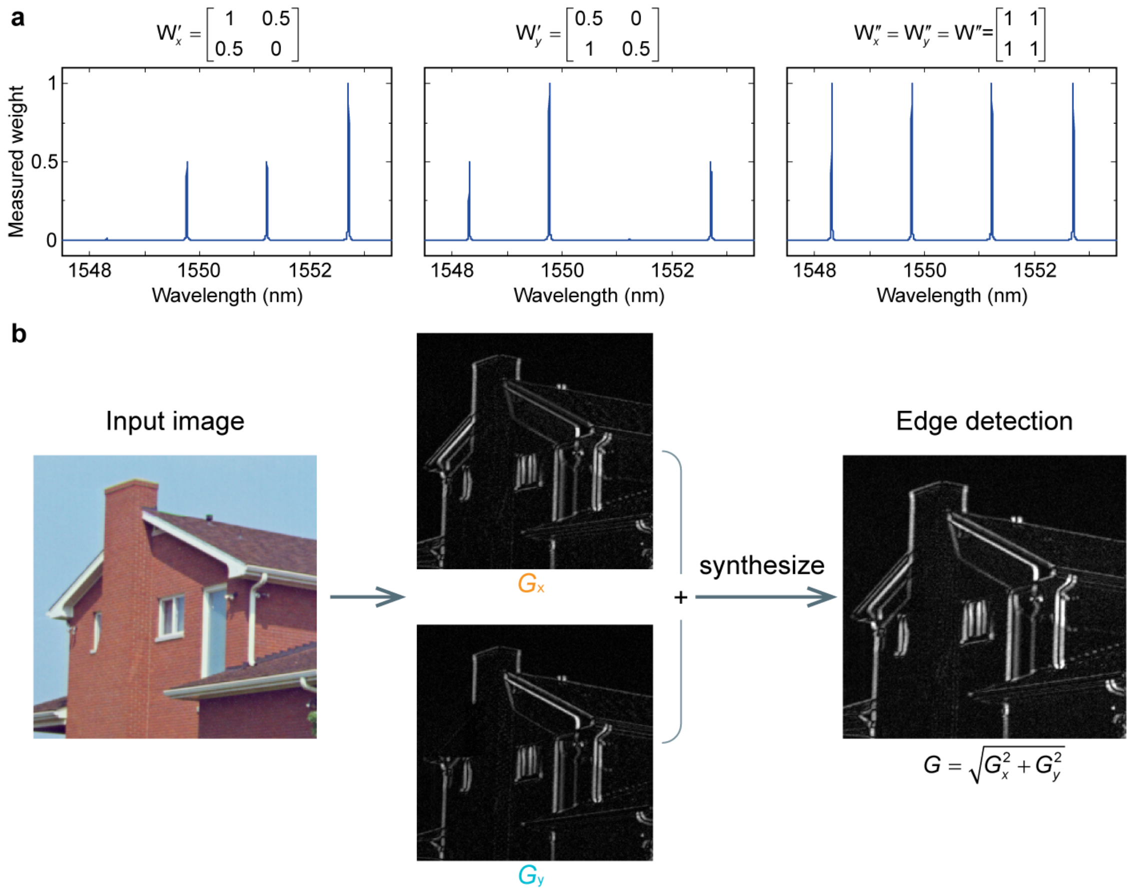

Fig. 4: a, The normalized spectrum of the microcomb lines when $\mathrm{W'}_x$**,** $\mathrm{W'}_y$ **and** $\mathrm{W''}$ **are implemented. b, Edge detection procedure using Roberts operator.**

The Robert operator was first mentioned by Lawrence Roberts in his thesis in 1963[3]. He believes that an operator should be able to produce a distinct edge that can be clearly perceived by the human eyes. In addition, other parts of the object and the background should not provide obvious intensity. Based on this, he proposed an operator that uses discrete gradients for edge detection. The operator is written as

$$\mathrm{W}_x = 2\mathrm{W'}_x - \mathrm{W''}_x = \begin{bmatrix} 1 & 0 \\ 0 & -1 \end{bmatrix} \text{ and } \mathrm{W}_y = 2\mathrm{W'}_y - \mathrm{W''}_y = \begin{bmatrix} 0 & -1 \\ 1 & 0 \end{bmatrix} \quad (6)$$

Fig. S4a shows the optical spectrum of the chip's output, corresponding to the kernels for matrices decomposition. When two operators slides over the image respectively, the

following results will be expressed as

$$\begin{cases} G_x = y_{i,j} - y_{i+1,j+1} \\ G_y = y_{i+1,j} - y_{i,j+1} \end{cases} \tag{7}$$

where i and j represent the image location. These results can be thought of as twice discrete gradient computation. Then, the edge detection results in both directions are combined with each other using the formula

$$G = \sqrt{G_x^2 + G_y^2}. \tag{8}$$

The experimental results of edge detection using Roberts operator is shown in Fig. S4b.

Supplementary note 6: Convolutional neural network training

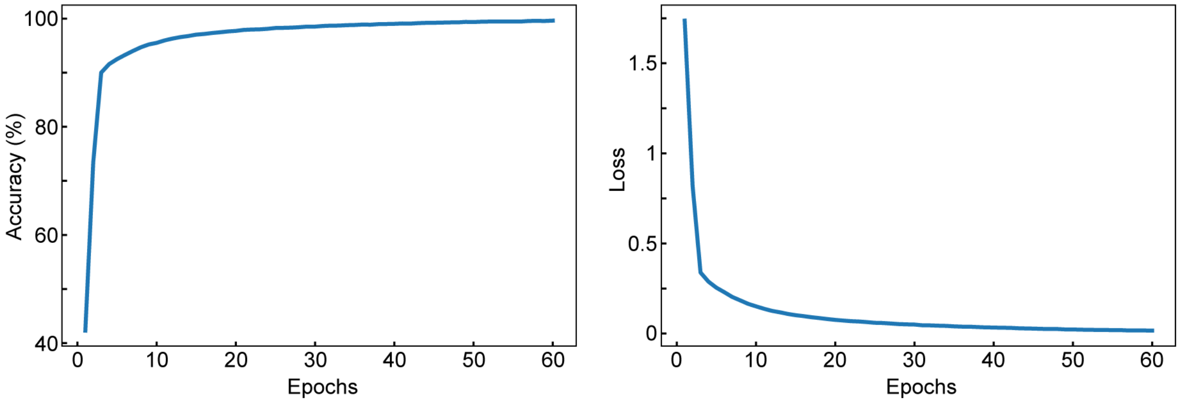

Fig. 5: Training of the convolutional neural network.

We use Pytorch, a neural network framework open sourced by Facebook(Meta), to train the neural network. Three kernels with size of 2×2 are used in the convolution layer. After the convolution layer, we add a fully connected layer with 512 neurons to process the feature map. The activation functions of the convolution layer and the fully connected layer are both the ReLu function. At the end of the neural network, we classify MNIST handwritten digits. Fig. S5 shows the curves of accuracy and loss function with the number of training epochs, where the loss function is the cross-entropy .

Supplementary note 7: Convolution procedure and prediction results for digits recognition

The digits recognition experiment implements the convolutional layer described in the main text (Fig. 4a). The microcomb is directly pumped by a DFB laser in the digits classification experiment. The comb lines when W'_1, W'_2 and W'_3 are loaded onto the MRR weight bank is shown in Fig. S6a. The procedure for digits recognition is shown in Fig. S6b. Since soliton microcombs co-integrated with a pump laser has revealed its viability[4–6], fully integrated microcomb with ultralow-noise by injection-locking a DFB laser is available in our future implement.

The input image are first arranged into the input vector with a size of 1×2916(27×27×4) and converts to RF differential signals by AWG. Then, the input RF signals are amplified by two linear electrical drivers and encoded on the intensity of the four comb lines via silicon EO modulator with a baud rate of 17 GBaud. Three 2×2 kernels are mapped to the MRR weight bank via matrix tuning controller and convolve with the input image as the comb lines pass through the chip. The convolution results in optical domain are collected by a photodetector connected to a oscilloscope. The experimentally obtained convolution results are sent to a computer. Alter ReLu activation, three feature maps are generated and then fed to fully connected layers which implemented electronically.

The detailed prediction results of the digits recognition is shown in Fig. S6c. Here, the confusion matrix is a 10×10 array computed for the test data set (500 handwritten digit pictures). Each cell $m(i, j)$ gives the percentage of number j classified into number i. In other words, the diagonal of the confusion matrix contains the correct classification produced by the photonic processing unit, while non-zero elements off the diagonal correspond to errors. The experimental implementation of the convolutional neural network reached an classification accuracy of 96.6%, showing good agreement with the calculated prediction accuracy of 97.0%.

To verify the operation of the MRR weight bank with data loaded, the electrical spectrum of the chip's input and output are given in Fig. S7a and Fig. S7b, respectively. Digits 0, 4, 8 are chosen as some examples. From the comparation, we can see that the experimental and calculated results for convolution with W, W′ and W″ are well conformed.

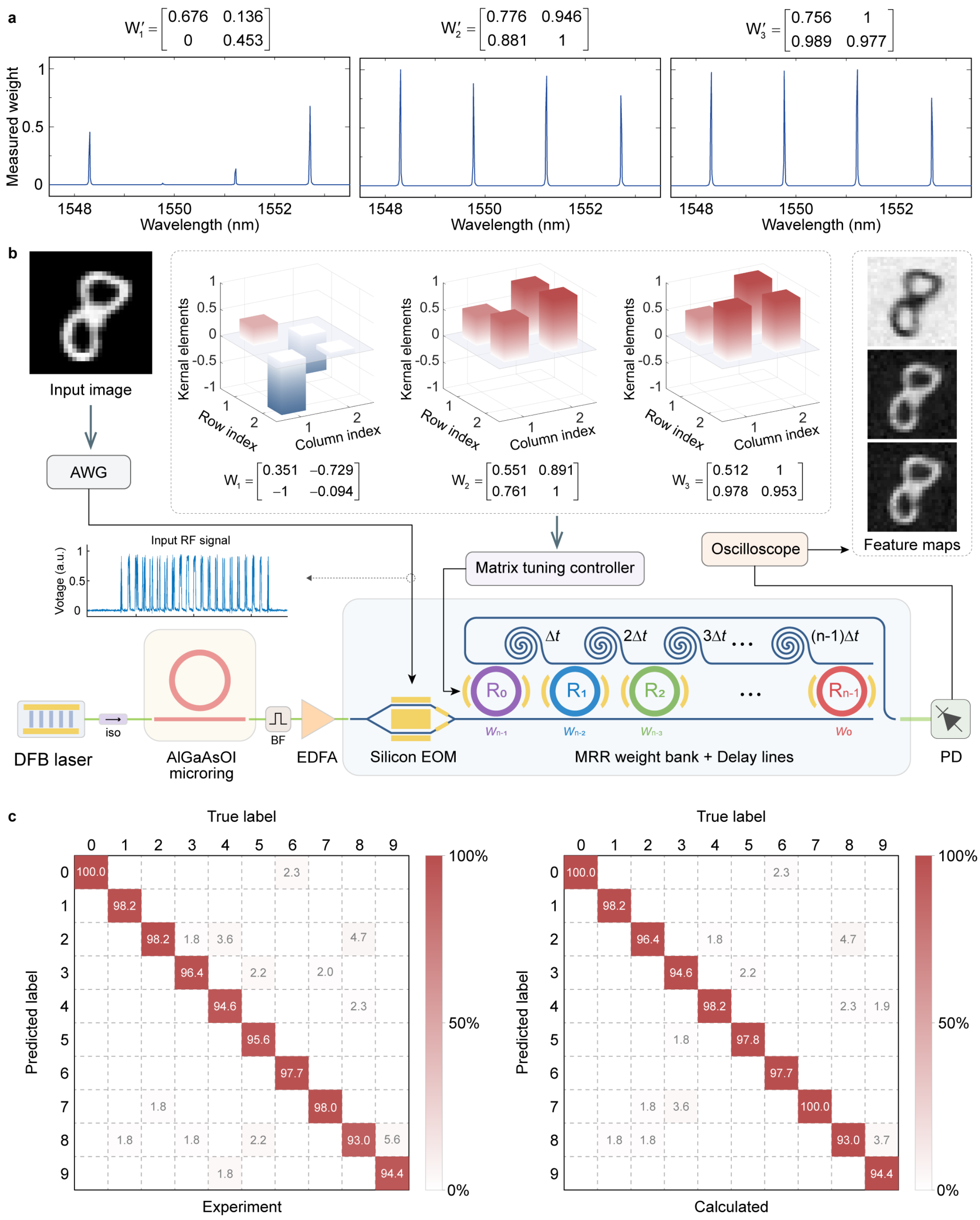

Fig. 6: Digits classification procedure and results. a, The normalized spectrum of the microcomb lines when W'_1, W'_1 and W'_3 are implemented. **b**, Experiment setup. **c**, Confusion matrices for the experimental and calculated convolutional neural networks.

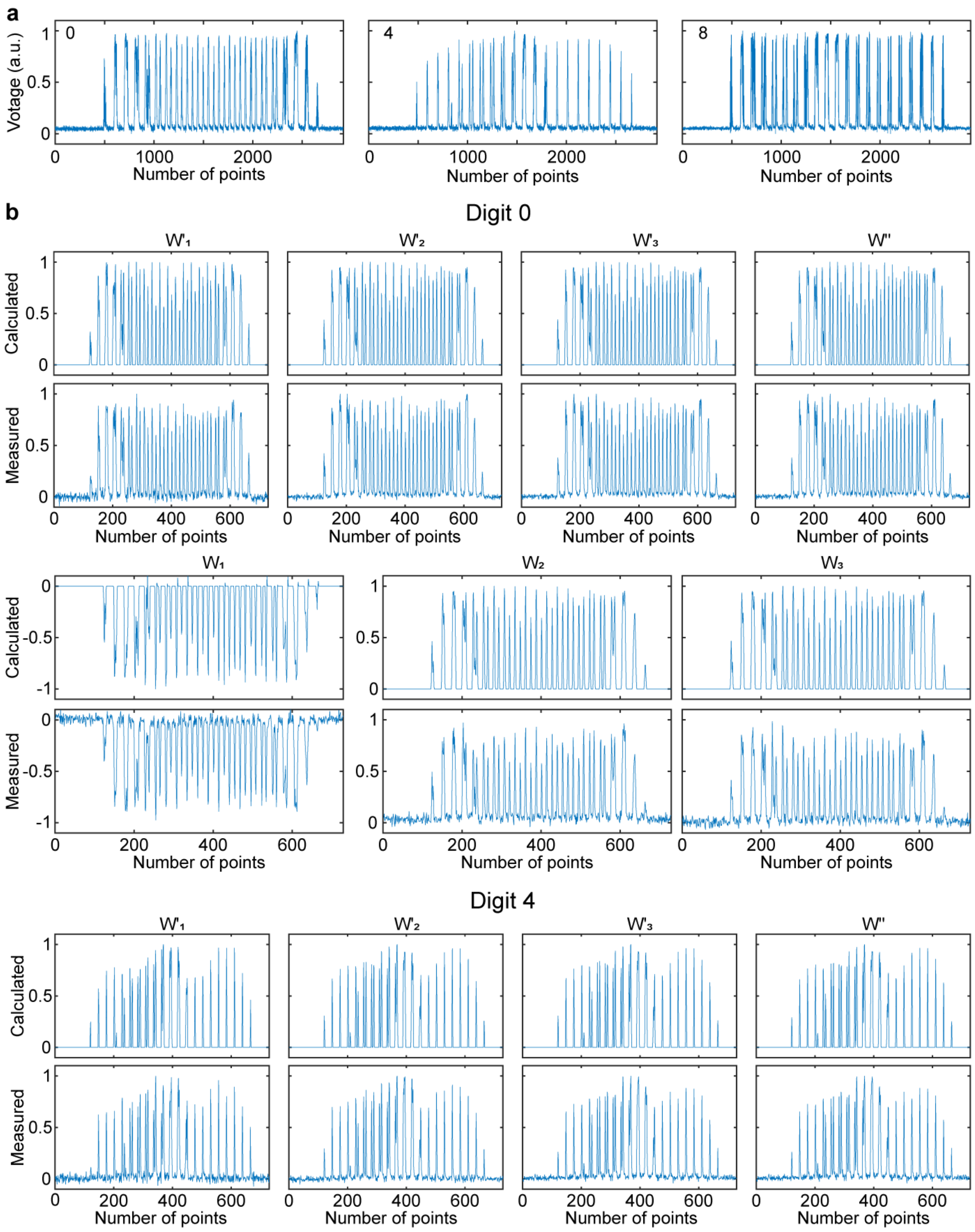
a
Votage (a.u.)
Number of points
b
Digit 0
W'_1
W'_2
W'_3
W''
W_1
W_2
W_3
Calculated
Measured
Digit 4

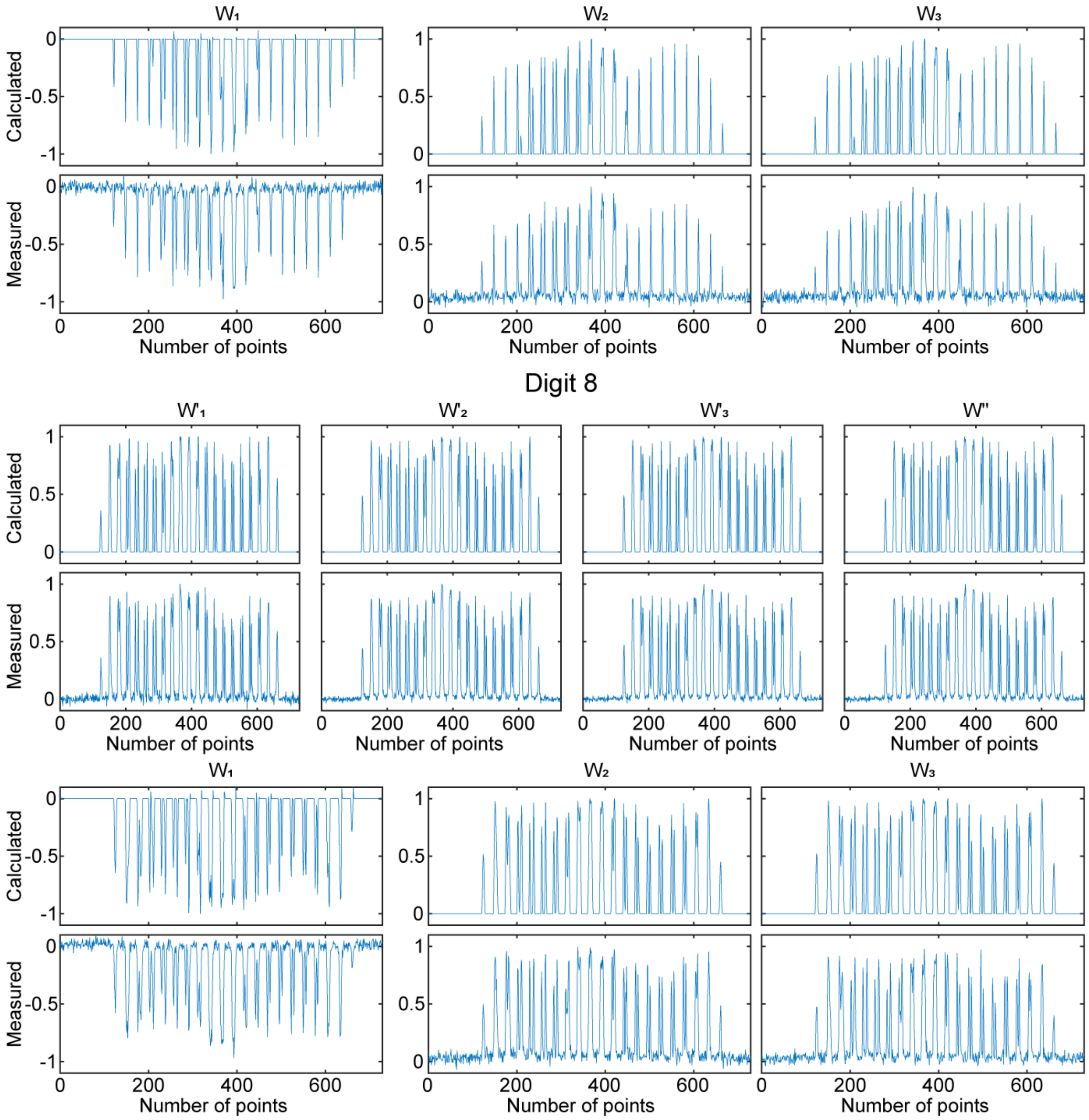

Fig. 7: Calculated and experimental waveforms. a, Input electrical spectrum. b, The calculated and measured results for convolution with W, W′ and W″.

Supplementary note 8: Compute density evaluation

To compare the performance of various photonic computing architectures, we define a figure-of-merit: *photonic core compute density* as follows[7]:

$$\text{Photonic core compute density} = \frac{\text{Computing speed (TOPS)}}{\text{Area of photonic linear operations unit (mm}^2\text{)}} \tag{9}$$

In this metric (also in line with the estimation protocols in ref[8, 9]), the photonic core compute density of our proof-of-concept PPU is 1.04 TOPS mm^{-2}. As the pump laser, microcomb source, silicon weight bank chip, and subsequent photo-detector are all essential components to perform convolution operations, here, we define another figure-of-merit: *overall compute density* as follows:

$$\text{Overall compute density} = \frac{\text{Computing speed (TOPS)}}{\text{Total area of the photonic chips (mm}^2\text{)}} \tag{10}$$

where the footprints of the mentioned above are all taken into consideration. To make a relatively comprehensive comparation, the detailed overall compute density estimation is shown in Table 1.

Table 1: Overall compute density estimation of the prototypical PPU

Components	Footprint (mm^2)	Overall compute density (TOPS mm^{-2})
DFB laser	1.2×0.33=0.396	$2\times4\times17\times10^9/1.311\approx0.104$
Microcomb	0.288×0.288≈0.083	
Weight bank chip	2.6×0.24+0.82×0.16≈0.755[a]	
Photo-detector[b]	0.28×0.275=0.077	
Total chip area	1.311	

[a] footprint of the silicon EOM and four MRRs with delay lines.

[b] replaced by a common on-chip Ge-Si photo-detector.

The footprints of DFB pump laser, comb source, silicon weight bank chip and photo-detector are all taken into consideration. The total photonic chip area is about 1.311 mm^2 and the corresponding overall compute density is about 0.104 TOPS mm^{-2}. Strictly speaking, the area of the digital processing unit (Intel(R) Core(TM) i7-10700K CPU) should be

included in the compute density estimation. However, the area of the digital chip and the area of each functional region (especially the computing core) have no public information that can be queried. Therefore, the area of the digital processing unit is not included in this estimation.

Table 2: Comparison of state-of-the-art integrated photonic computing hardwares

Technology	Data loading rate (Gbaud)	Weight precision (bits)	Energy efficiency (TOPS W^{-1})	Compute density[a] (TOPS mm^{-2})
MZI mesh[10]	–	8	–	–
MZI mesh[11]	1×10^{-5}	4	–	9.88×10^{-7}
Cascaded MZI[12]	1×10^{-7}	6.6	–	–
InP SOA[13]	10	∼4.5	∼0.24	–
Diffrative cell[14]	$\sim1\times10^{-5}$	–	∼0.11	$\sim3.77\times10^{-3}$
MRRs[15][b]	0.047	>5.5	–	–
Photonic neuron[9][b]	N/A	–	∼0.07	3.5
PCM+Waveguide[16]	1×10^{-6}	6	–	2.5×10^{-6}
WDM+PCM[8]	2	5	0.4	0.2
This work	17	9	$0.2/1.52\times10^{-3}$ [c]	1.04/0.104[d]

[a] only the photonic linear operation area is considered for all works.

[b] contains multiple layers.

[c] refers to expected/current energy efficiency.

[d] refers to photonic-core/overall compute density.

A comparison among the representative integrated photonic computing hardware is summarized in Table 2. Due to the high data loading rate and the associated calibration procedure, the photonic core compute density in this work is over 1 TOPS mm^{-2} and a record high weight control precision of 9 bits is achieved. Although the overall compute density of the prototypical PPU is small, it can be further improved by increasing the modulation speed and reducing the chip area. For instance, using a monolithic integrated microcomb source[17] and replacing the silicon modulator with ultra-compact hybrid plasmonic Mach–Zehnder modulator[18], the total chip area can be dramatically reduced.

Supplementary note 9: Energy efficiency estimation

The power consumption of the current PPU are mainly from six aspects: InP DFB pump laser, EDFA, silicon photonic chip, modulator drivers, TEC and digital backend, as listed in table 3.

Table 3: Estimated power consumption of the proof-of-concept PPU

Components	Voltage (V)	Current (A)	Power (W)
DFB laser	1.500	0.300	0.450
Silicon photonic chip			
DC bias1	2.332	0.047	0.109
DC bias2	1.697	0.034	0.058
Thermal phase shifter1	0	0	0
Thermal phase shifter2	2.500	1.25×10^{-2}	0.031
On-ring heaters	$\bar{V} \approx 2$	$\sim 5 \times 10^{-3}$	$\sim$0.01×4=0.04
EDFA	N/A	0.860	$\sim 50^a$
Modulator driver1	9.000	0.275	2.475
Modulator driver2	9.000	0.258	2.322
TEC for DFB laser	1.830	0.680	1.243
TEC for microcomb	1.372	0.078	0.107
TEC for Silicon photonic chip	5.920	0.450	2.664
CPU			$\sim 30^b$
Total power consumption			~ 89.5

[a] typical power consumption according to the user manual.

[b] from a power monitor software[19] when implementing fully-connected layers.

For the Silicon photonic chip, the DC bias represents the reverse bias voltage for depleted PN junction of the Si modulator; The thermal phase shifters are used to adjust the demanded operating status for DC bias-point of the EO modulator; The kernel weights are tuned

by the 4 on-ring heaters. The power consumption information of the CPU to implement fully-connected layers is directly obtained from a power consumption monitor software[19]. The total current power consumption is calculated as about 89.5 W, including the power consumption of the EDFA and digital backend.

Benefit from the recent advances in integrated optical frequency combs[4, 20], circuit-based erbium-doped amplifier[21], on-chip semiconductor optical amplifiers (SOA)[22] and the mature on-chip filter technology, the discrete EDFA, band-pass filter can be replaced by fully integrated photonic devices eventually. Furthermore, with the development of hybrid and monolithic integration technology, the light source, silicon photonic circuit and the associated electronic blocks(including modulator drivers, transimpedance amplifiers (TIAs), digital-to-analog converters (DACs), analog-to-digital converters (ADCs)) can be all integrated in a same main-board or even in a single chip.

To show the ultimate potential of our architecture, the excepted power consumption calculated in line with similar protocols in[8, 23] is also provided here. The on-chip pump power to generate the microcomb can be as low as 98 mW[4]. When using low-loss phase change materials[24], the power consumption of the MRR weight bank could be "near zero". The typical power consumption of the on-chip SOA[22] is 390 mW. The energy consumption from photodetection is dominated by the transimpedance amplifier (TIA). The power cost from the assorted digital circuits: 5.36 pJ Sa^{-1} driver (28 GHz)[25], 1.14 pJ Sa^{-1} TIA (53 GHz)[26], 2.72 pJ $conversion^{-1}$ DAC (8 bits)[27], 2 pJ $conversion^{-1}$ ADC (8 bits)[28]. If utilizing high order MRRs[29] combined with MZI-embedded microring[30], the flat-top pass band enable high modulation rate and the weight tuning can be realized by the embedded MZI. The computing speed of the prototypical PPU is 0.136 TOPS and the expected power consumption is $98+390+(5.36+1.14+2.72+2)\times17=678.74$ mW. The expected energy efficiency of our prototypical PPU is 0.136 TOPS/(678.74 mW) $\approx$ 0.2 TOPS W^{-1}.

For a PPU with 5×5 kernel matrix size, if the modulation rate is 50 Gbaud, the computing speed of the PPU will be promoted to $50\times10^9\times25\times2=2.5$ TOPS. The corresponding expected power consumption will be $98+390+(5.36+1.14+2.72+2)\times50=1049$ mW=1.049 W. Then, the expected energy efficiency will be 2.5 TOPS/(1.049 W) $\approx$ 2.38 TOPS W^{-1}. It should be noted that the expected power consumption estimation is based on ideal conditions. The performance of different electronic blocks may not the same if implemented on a single processor and many factors (such as thermal, coupling, interference, etc.) can lead to

an increase in the power consumption once they are packaged together. Nevertheless, compared to its electronic competitors (e.g. CPU and GPU), the potential energy consumption advantage of the PPU is still significant.

[1] Tait, A. N. *et al.* Feedback control for microring weight banks. *Optics express* **26**, 26422–26443 (2018).

[2] Tang, Z., Pan, S. & Yao, J. A high resolution optical vector network analyzer based on a wideband and wavelength-tunable optical single-sideband modulator. *Optics express* **20**, 6555–6560 (2012).

[3] Roberts, L. G. *Machine perception of three-dimensional solids.* Ph.D. thesis, Massachusetts Institute of Technology (1963).

[4] Stern, B., Ji, X., Okawachi, Y., Gaeta, A. L. & Lipson, M. Battery-operated integrated frequency comb generator. *Nature* **562**, 401–405 (2018).

[5] Raja, A. S. *et al.* Electrically pumped photonic integrated soliton microcomb. *Nature communications* **10**, 1–8 (2019).

[6] Shen, B. *et al.* Integrated turnkey soliton microcombs. *Nature* **582**, 365–369 (2020).

[7] Nahmias, M. A. *et al.* Photonic multiply-accumulate operations for neural networks. *IEEE Journal of Selected Topics in Quantum Electronics* **26**, 1–18 (2019).

[8] Feldmann, J. *et al.* Parallel convolutional processing using an integrated photonic tensor core. *Nature* **589**, 52–58 (2021).

[9] Ashtiani, F., Geers, A. J. & Aflatouni, F. An on-chip photonic deep neural network for image classification. *Nature* **606**, 501–506 (2022).

[10] Shen, Y. *et al.* Deep learning with coherent nanophotonic circuits. *Nature Photonics* **11**, 441–446 (2017).

[11] Zhang, H. *et al.* An optical neural chip for implementing complex-valued neural network. *Nature Communications* **12**, 1–11 (2021).

[12] Xu, S. *et al.* Optical coherent dot-product chip for sophisticated deep learning regression. *Light: Science & Applications* **10**, 1–12 (2021).

[13] Shi, B., Calabretta, N. & Stabile, R. Deep neural network through an inp soa-based photonic integrated cross-connect. *IEEE Journal of Selected Topics in Quantum Electronics* **26**, 1–11 (2019).

[14] Zhu, H. *et al.* Space-efficient optical computing with an integrated chip diffractive neural network. *Nature Communications* **13**, 1–9 (2022).

[15] Huang, C. *et al.* A silicon photonic–electronic neural network for fibre nonlinearity compensation. *Nature Electronics* **4**, 837–844 (2021).

[16] Wu, C. *et al.* Programmable phase-change metasurfaces on waveguides for multimode photonic convolutional neural network. *Nature communications* **12**, 1–8 (2021).

[17] Xiang, C. *et al.* Laser soliton microcombs heterogeneously integrated on silicon. *Science* **373**, 99–103 (2021).

[18] Haffner, C. *et al.* All-plasmonic mach–zehnder modulator enabling optical high-speed communication at the microscale. *Nature Photonics* **9**, 525–528 (2015).

[19] HWiNFO. Professional system information and diagnostics. `https://www.hwinfo.com/` (2022).

[20] Chang, L., Liu, S. & Bowers, J. E. Integrated optical frequency comb technologies. *Nature Photonics* **16**, 95–108 (2022).

[21] Liu, Y. *et al.* A photonic integrated circuit–based erbium-doped amplifier. *Science* **376**, 1309–1313 (2022).

[22] Thorlabs, Inc. Saf1126c c-band saf gain chip. `https://www.thorlabs.com/newgrouppage9.cfm?objectgroup_id=3944` (2022).

[23] Xu, X. *et al.* 11 tops photonic convolutional accelerator for optical neural networks. *Nature* **589**, 44–51 (2021).

[24] Li, T. *et al.* Nonvolatile switching in in2se3-silicon microring resonators. In *CLEO: Science and Innovations*, SM4B–5 (Optical Society of America, 2021).

[25] Temporiti, E. *et al.* 23.4 a 56gb/s 300mw silicon-photonics transmitter in 3d-integrated pic25g and 55nm bicmos technologies. In *2016 IEEE International Solid-State Circuits Conference (ISSCC)*, 404–405 (IEEE, 2016).

[26] Lakshmikumar, K. R. *et al.* A process and temperature insensitive cmos linear tia for 100 gb/s/¡inline-formula¿ ¡tex-math notation="latex"¿λ ¡/tex-math¿¡/inline-formula¿ pam-4 optical links. *IEEE Journal of Solid-State Circuits* **54**, 3180–3190 (2019).

[27] Kossel, M. A. *et al.* 8.3 an 8b dac-based sst tx using metal gate resistors with 1.4pj/b efficiency at 112gb/s pam-4 and 8-tap ffe in 7nm cmos. In *2021 IEEE International Solid- State Circuits Conference (ISSCC)*, vol. 64, 130–132 (2021).

[28] Kull, L. *et al.* A 24-to-72gs/s 8b time-interleaved sar adc with 2.0-to-3.3pj/conversion and gt;30db sndr at nyquist in 14nm cmos finfet. In *2018 IEEE International Solid - State Circuits*

Conference - (ISSCC), 358–360 (2018).

[29] Xia, F., Rooks, M., Sekaric, L. & Vlasov, Y. Ultra-compact high order ring resonator filters using submicron silicon photonic wires for on-chip optical interconnects. *Optics express* **15**, 11934–11941 (2007).

[30] Chen, B. *et al.* Silicon-based mzi-embedded microring array with hitless and fsr-alignment-free wavelength selection. *IEEE Photonics Technology Letters* **34**, 436–439 (2022).
